# Copper-containing nanoparticles: Mechanism of antimicrobial effect and application in dentistry-a narrative review

**DOI:** 10.3389/fsurg.2022.905892

**Published:** 2022-08-05

**Authors:** Xinru Ma, Shiyu Zhou, Xiaoling Xu, Qin Du

**Affiliations:** ^1^ Department of Stomatology, Sichuan Provincial People’s Hospital, University of Electronic Science and Technology of China, Chengdu, China; ^2^ Department of Stomatology, Hospital of Chengdu Office of People's Government of Tibetan Autonomous Region (West China Hospital Sichuan University Tibet Chengdu Branch Hospital), Chengdu, China; ^3^ School of Materials Science and Engineering, Southwest Jiaotong University, Chengdu, China

**Keywords:** copper nanoparticles, antimicrobial effect, oral pathogen, streptococcus mutans, porphyromonas gingivalis, candida albicans

## Abstract

Copper has been used as an antimicrobial agent long time ago. Nowadays, copper-containing nanoparticles (NPs) with antimicrobial properties have been widely used in all aspects of our daily life. Copper-containing NPs may also be incorporated or coated on the surface of dental materials to inhibit oral pathogenic microorganisms. This review aims to detail copper-containing NPs’ antimicrobial mechanism, cytotoxic effect and their application in dentistry.

## Introduction

Copper is a common metal with unique physical and chemical properties. Copper is the 26th most abundant element in the Earth's crust (1). Copper has been used in coins, jewelry, and utensils since ancient times, and the potential of copper to promote health effects was recognized as early as 3,000 BC (2, 3). A large variety of dental restorative materials contain copper, such as dental amalgam, porcelain-fused-to-metal crowns, implants, and partial denture attachments and frameworks (4–7). Copper is an essential trace element for humans and can promote angiogenesis, bone formation, wound healing, and the activities of various enzymes (8–10). Additionally, it also catalyzes the formation of crosslinks in collagen and elastin precursors (11–13). Moreover, copper is essential for maintaining the normal physiological functions of microorganisms. But high concentrations of copper can be used as microbicides (14–16). Prior to the development of antibiotics, inorganic antibacterial agents, such as silver and copper, were used to treat microbial infections (17). The paper reported copper as an antimicrobial coating as early as 1962 (18). Also, current research has shown that copper has a much less toxic effect on mammalian cells than silver (19).

With the progress of nanotechnology, copper has been increasingly used in the medical field, such as the latest copper-containing Nanoparticles (NPs), which have been proven to inhibit a variety of oral microorganisms, such as *Streptococcus mutans* (*S. mutans*) (20–22), *Porphyromonas gingivalis* (*P. gingivalis*) (23), and *Candida albicans* (*C. albicans*) (20, 24–26). In 2008, the International Organization for Standardization (ISO) defined NPs as discrete nano-objects with all three external dimensions less than 100 nm. In 2011, the European Commission set a more technical but wider ranging definition: a natural, incidental, or manufactured material containing particles in an unbound state, as an aggregate, or as an agglomerate and where, for 50% or more of the particles in the number size distribution, one or more external dimensions is within the size range 1–100 nm. Under this definition, nanomaterials can be classified as NPs only if one of their characteristic dimensions is within the range of 1–100 nm. NPs have many unique physical and chemical properties, such as tunable size, biocompatibility, and singlet oxygen generation, which allows them to be widely used (27, 28).

In recent years, the application of nanomaterials in dentistry has gradually increased, and copper NPs can be used as a new type of antimicrobial material (28). This paper reviews the antimicrobial mechanisms of copper-containing NPs and their application in dentistry.

## Different types of copper-containing NPs

Various types of copper-containing NPs are successfully synthesized, such as copper NPs (Cu NPs), copper oxide NPs (Cu_x_O NPs), and copper-containing bimetallic NPs. Cu_x_O NPs are widely used in the fields of biomedicine, environmental restoration, and industry (29–31). In biomedicine, cuprous oxide (Cu_2_O) and cupric oxide (CuO) are often used as antimicrobial agents (32–34). Compared with organic antimicrobial agents, copper oxide has the advantages of stable physical and chemical properties, solidity, and a relatively long shelf life. Moreover, copper oxide has physical properties that allow it to be easily mixed with polymers, which enables Cu_x_O NPs to be prepared into a variety of composite materials. Compared with Cu_x_O NPs, Cu NPs are relatively unstable and easily oxidized. Copper (Cu) is easily oxidized to form Cu_2_O and CuO when exposed to the air, making it difficult to synthesize Cu NPs in an ambient environment. Therefore, it is usually necessary to synthesize Cu NPs in the presence of polymers and surfactants and form coatings on the surface of Cu NPs (25). Copper-containing bimetal NPs are NPs containing copper and another metal element. The combination of two metal elements will have a synergistic effect and may have better antimicrobial performance than single metal. For example, Perdikaki et al. (35) have shown that synthesized Ag/Cu bimetallic NPs have stronger antimicrobial properties than Ag and Cu monometallic NPs.

These different types of copper-containing NPs can be incorporated into supporting materials (chitosan, cellulose polymers, hydrogels, *etc*.), which are biocompatible and retain antimicrobial activity (36–40). Tran CD et al. (41) synthesized composites containing cellulose, chitosan, and CuO NPs. This composite can prevent the aggregation, coagulation, and changes in size and morphology of CuO NPs without changing the unique properties of the NPs. Moreover, they can exert superior antibacterial activity against a variety of bacteria and fungi, and the antibacterial activity is related to the content of CuO NPs. As chitosan is a biocompatible, biodegradable, and non-toxic polymer, copper-containing NPs can be incorporated into chitosan and used in dental materials. Chitosan can interact with hydroxyapatite and the bacterial cell walls of teeth to improve the adhesion of copper on the tooth surface and the anti-biofilm action of copper (42). Chitosan not only has a good inhibitory effect on Gram-negative bacteria, Gram-positive bacteria, and fungi (43), but also interferes with oral microbial adhesion, inhibit biofilm formation and maturation, and promote wound and oral ulcer healing (43–46). Mishra et al. (47) synthesized biocompatible thiol-functionalized cellulose-grafted copper oxide nanoparticles, which alleviated colitis conditions and recovered damaged colon structure. Cellulose enhances the biocompatibility of copper oxide NPs and avoids the adverse effects of CuO NPs on the biological systems. The Cu-NP-embedded hydrogels also possessed remarkable antibacterial ability, and reduced the inflammatory response and promoted angiogenesis *in vivo* to accelerate the wound healing process (48). By preparing copper-containing NPs and other materials into composites, the original physical and chemical properties of copper-containing NPs can be retained while giving composites new characteristics, making them more suitable for clinical application.

## Antimicrobial mechanism of copper-containing NPs

Copper can cause damage to various cell functions and exert cytotoxicity, making it an effective microbial inhibitor. In general, copper damages microbial cells by generating reactive oxygen species (ROS) and replacing or binding the native cofactors in metalloproteins (49). Besides, copper is also involved in innate immunity and can catalyze the formation of ROS in the blasting reaction taking place within phagocytes, enhancing the bactericidal activity during bacterial phagocytosis (14, 50).

Copper-containing NPs can inhibiting microorganisms through the same mechanism as other types of copper materials mentioned above (51–53). Many studies have shown that NPs can exert stronger antimicrobial properties than ordinary size materials, but the reason for this is not completely clear at present. Compared with other copper molecular materials, Copper-containing NPs has higher surface area and different crystal structure, and can affect different cellular components of microbial cells through some unique mechanisms to exert better antibacterial activity (54–59). Copper-containing NPs can dissolve faster in solutions, release more metal ions, and exert a stronger antimicrobial effect (60). In addition, Copper-containing NPs can bring multiple antibacterial mechanisms simultaneously, but it is difficult for the same microorganism to have multiple gene mutations to cope with various antimicrobial mechanisms of NPs, so the probability of antimicrobial resistance is low.

**Figure 1:**
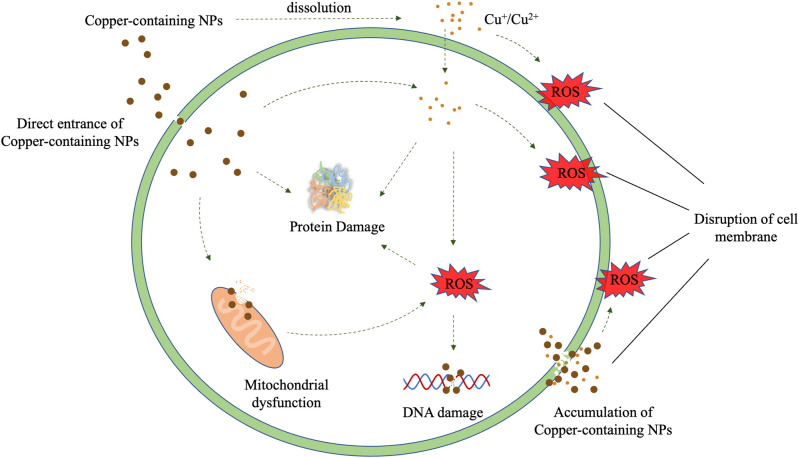
Illustration of possible antibacterial mechanism of Copper-containing NPs.

In general, Copper-containing NPs added to many dental materials inhibits microorganisms mainly through the release of the NPs and copper ions. The antimicrobial process of copper-containing NPs is to produce ROS, destroy cell walls and cell membranes, and react with proteins and DNA (61). In this process, copper-containing NPs can damage different microbial cell components through a variety of mechanisms (Figure 1).

### Generation of ROS

Oxidative stress caused by ROS is crucial in the antibacterial effect of copper. ROS are oxygen-containing derivatives composed of highly unstable oxygen radicals, such as superoxide (O_2_^•−^), hydroxyl (OH^•^), hydrogen peroxide (H_2_O_2_), and singlet oxygen (O_2_) (62). The atomic or molecular orbitals of ROS contain one or more unpaired electrons, which makes them highly reactive (63). Transition metals, such as copper, iron, and silicon, can generate ROS through Fenton type and Haber-Weiss reactions:

$$\begin{array}{c} Cu^{+}+O_{2}\to Cu^{2+}+O_{2}^{\cdot -},2O_{2}^{\cdot -}+2H^{+}\to H_{2}O_{2}\\ +O_{2},Cu^{+}+H_{2}O_{2}\to Cu^{2+}+\mathrm{OH}\cdot +OH^{-} \end{array}$$

During these reactions, copper accepts and donates an electron during cycling between the Cu^+^ and Cu^2+^ oxidation states, producing O_2_^•−^ and hydroxyl OH^•^, which are highly reactive and have strong damaging potential, leading to lipid peroxidation, protein oxidation, and DNA damage (64–66).

In the presence of water and oxygen molecules, copper can only dissolve a small amount of copper ions (67). In fact, the metal ions released by dissolution outside the cell are not the main antibacterial mechanism of copper-containing NPs. A recent study showed that dissolved copper ions contributed less than half of the total cytotoxicity induced by CuO NPs (68). A possible reason for this is that metal-based NPs enter the acidic lysosomal environment (pH 5.5) in cells, which promotes the formation of free radicals and the degradation/corrosion of NPs, thereby converting the core metal into an ionic form and resulting in the intracellular release of metal ions. This process of the internalization of NPs is often referred to as “the Trojan horse mechanism”, which promotes the formation of intracellular ROS (69, 70). The formation of ROS is an important part of the antimicrobial mechanism of copper-containing NPs. Copper-containing NPs can not only generate ROS by directly leaching ions but may also generate ROS through multiple mechanisms, such as the disruption of mitochondrial membrane potential, and produce singlet oxygen to mediate the degradation of DNA (71, 72). However, these mechanisms have not been fully clarified, and relevant microbial cytotoxicity studies are lacking.

### Disruption of microbial cell walls and cell membranes

The early stage of copper-containing NPs damage to microbials is their direct contact with the microbial surface, which leads to the alteration of the microbial cell wall and cell membrane (73). Metal-based NPs and their leached metal ions are positively charged, and the surface of both Gram-positive and Gram-negative bacteria are negatively charged. Therefore, through electrostatic interaction, metal-based NPs will be adsorbed onto the surface of bacteria and build strong bonding leads for the destruction of the cell wall. This process increases cell permeability and allows metal-based NPs to enter the cell easily (74). Besides, copper ions can be combined with negatively charged areas on the cell membrane to reduce the potential difference and cause depolarization. When the potential difference drops to zero, it will cause membrane leakage or even rupture, exposure of the cellular components, and, eventually, bacterial death (75). Many studies have shown that the cell membrane is a direct target of copper exposure (76, 77). Hong et al. (78) found that *E. coli* died after 45 min of copper alloy contact, but no degradation of genomic DNA was observed. Copper ions can cause oxidative damage to the unsaturated fatty acids of bacterial cell membrane phospholipids through the production of extracellular ROS, while OH· can drive the non-enzymatic peroxidation of the unsaturated double bonds of fatty acids, thereby triggering a series of reactions and leading to extensive changes in the structure of the phospholipid bilayer and destroying the biophysical properties of the membrane, which ultimately leads to a loss of membrane integrity, exposure of the cell components, and cell death.

However, the bactericidal effect of copper-containing NPs on Gram-positive bacteria is stronger than that of Gram-negative bacteria, which may be due to the difference in the cell wall structure of these two classes. Compared with lipids, copper has a higher affinity for proteins, so Gram-positive bacteria with higher levels of peptidoglycan and protein content in the cell wall is more easily destroyed by copper-containing NPs (44).

### Replace or bind the native cofactors in metalloproteins

Previous studies believed that copper toxicity was mainly related to the production of ROS, but later studies found that, under anaerobic conditions, copper accumulation can also increase cytotoxicity to bacteria (79, 80). Moreover, recent studies have shown that copper's cytotoxicity to microorganisms is also closely related to its ability to replace or bind to the native cofactors in metalloproteins. Intracellular copper accumulation promotes mismetallation, which is mainly related to the iron-sulfur cluster protein and its assembly process (81). Specifically, the copper accumulated in bacterial cells mainly exists in the form of highly toxic Cu^+^, which coordinates with the thiolate or inorganic sulfur ligands of the solvent-exposed dehydratase and replaces the iron atom, rapidly inactivating Fe/S cluster dehydratases to cause cell dysfunction (82, 83). In addition, copper and iron in *Escherichia coli* (*E. coli*) cells seem to share the same binding site in the Iron-sulfur cluster assembly protein (IscA), and excessive copper can also compete with iron for metal binding sites in IscAs and effectively inhibit the IscA-mediated assembly of [4Fe-4S] clusters (81, 84).

### Damage of intracellular components

As described above, the uniqueness of the toxicity of copper-containing NPs to microorganisms is due to that they can directly enter the cells, and are internalized into complete intracellular particles in microbial cells through the Trojan horse mechanism. Studies by Kaweeteerawat et al. (85) showed that, at low concentrations, copper ions mainly inhibit microorganisms by damaging cell membranes rather than by causing oxidative stress in cells. However, they found that, even at lower concentrations, copper-containing NPs are also sufficient to promote the production of large amounts of intracellular ROS. In general, copper-containing NPs entering cells can directly damage oxidative organelles, such as mitochondria, and lead to increased intracellular ROS, protein oxidation, and DNA degradation (86, 87). Studies by Chatterjee et al. (87) showed that the oxidation of proteins in cells is mediated by ROS, but the degradation of DNA is a ROS-independent phenomenon caused by the intracellular release of copper ions. Studies by Giannousi et al. (33) have also found that copper-containing NPs induce DNA degradation in a dose-dependent manner and extensively degrade double-stranded calf thymus DNA (dsCT-DNA) at low concentrations. In general, the exact mechanism of the antimicrobial effect of copper-containing NPs is unclear and needs to be elucidated.

## Factors affecting antimicrobial effect

The main physical factors affecting the antimicrobial activity of copper-containing NPs are include the size, morphology and environmental conditions (temperature) of the NPs., Chemical factors include environmental conditions (PH value, dry or wet, and composition of the surrounding medium), the doping modification of other elements, and the oxidation state of copper.

### Size and morphology

It has been suggested that due to the small size and high surface-to-volume ratio, metallic NPs can exert better antimicrobial activity than ordinary metals (88–91). At similar surface area doses, copper NPs and copper microparticles have similar effects on cell membrane damage, reflecting the fact that the damage of the cell membrane is related to the surface area of NPs (60, 89). Different sizes of copper-containing NPs have different inhibitory effects on Gram-positive and Gram-negative bacteria (92). Azam et al. (93) found that the small size CuO NPs is more stable than the large size CuO NPs and has significantly stronger antibacterial properties. Some studies have also proved that the antibacterial activity of CuO NPs and Cu_2_O NPs are size-dependent: the reduction in the size of the NPs leads to an increase in antibacterial properties (93–95). Applerot et al. (94) believed that the reason for the stronger antibacterial effect of small-size CuO NPs is due to their stronger ability to penetrate cells. The high surface-to-volume ratio and small size of copper-containing NPs enhance their interaction with microbial membranes, enabling them to exert stronger antimicrobial activity than copper ions.

**Figure 2:**
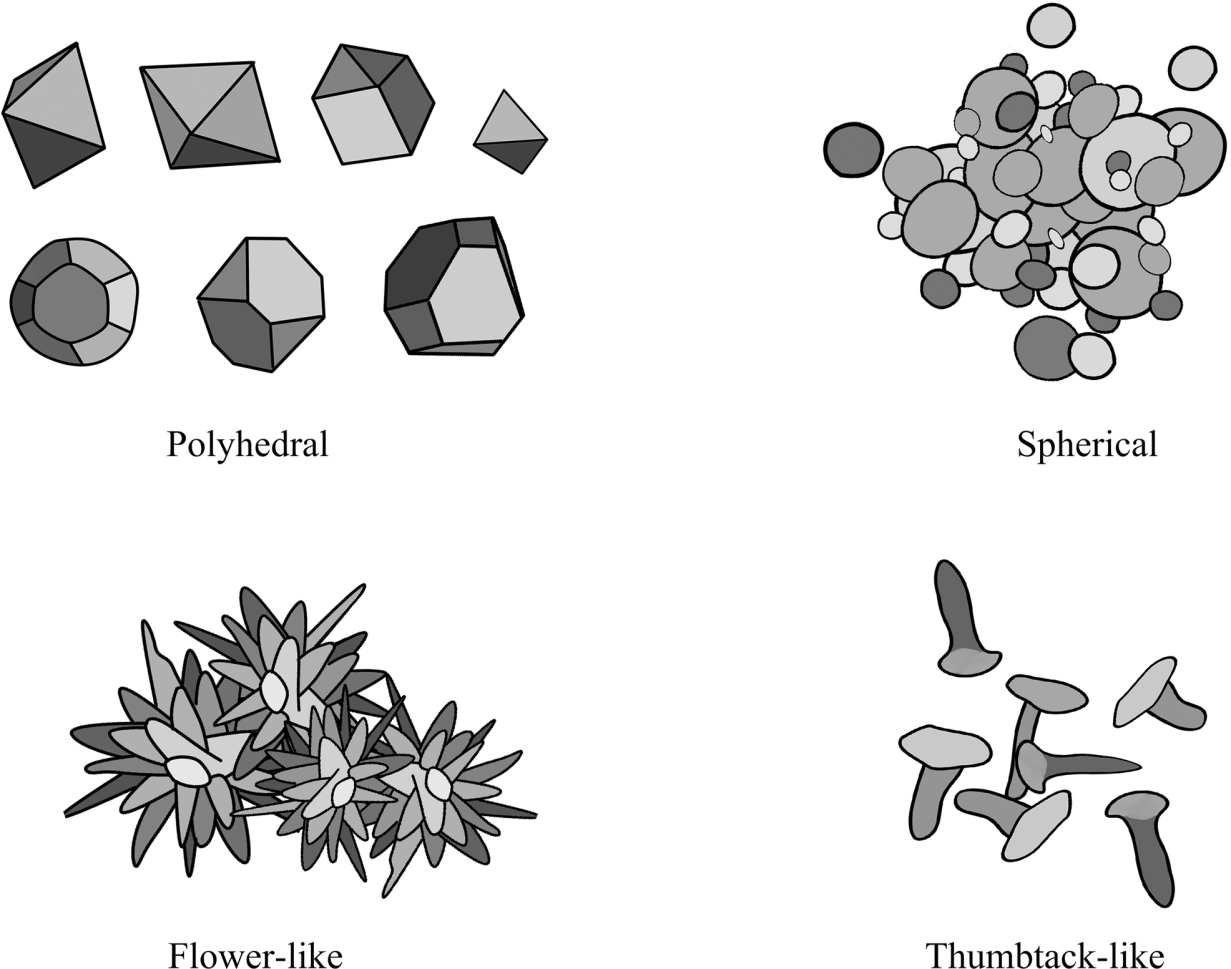
Different morphology of Copper-containing NPs.

The antimicrobial activity of NPs is also related to morphology, and different morphologies of NPs can cause different degrees of bacterial cell damage through interactions with periplasmic enzymes (96). Copper-containing NPs with different crystal planes have different surface energies, and this difference may also be responsible for the morphologically dependent antimicrobial activity of copper-containing NPs. The higher surface energy of the exposed facets of copper-containing NPs may generate copper ions more effectively and lead to stronger antimicrobial activity (97, 98). Xiong et al. (99) synthesized polyhedral, ﬂower-like, and thumbtack-like Cu/Cu_x_O NPs. And they proved that, among the three kinds of Cu / Cu_x_O NPs, the main exposed facets {111} of the ﬂower-like Cu / Cu_x_O NPs had the highest surface energy, dissolved the most copper ions in the culture medium, and had the best antibacterial ability. Studies by Feng et al. (100) have shown that {100} facets of the Cu_2_O nanocrystals can release more copper ions and produce more ROS in a shorter amount of time than {111} facets of the Cu_2_O nanocrystals, resulting in stronger toxicity in the short term. Besides, some studies believe that {110} facets of the Cu_2_O microcrystals have better antibacterial activity against *E. coli* than that of {111} facets (101, 102). However, on the contrary, some studies also believe that {111} facets of the Cu_2_O microcrystals have stronger antibacterial properties (103). In addition, studies by Pang et al. (97) have shown that the antibacterial activity of cubic Cu_2_O has a broad spectrum, while the antibacterial activity of octahedral Cu_2_O has high selectivity (Figure 2).

### Ambient conditions

**Figure 3:**
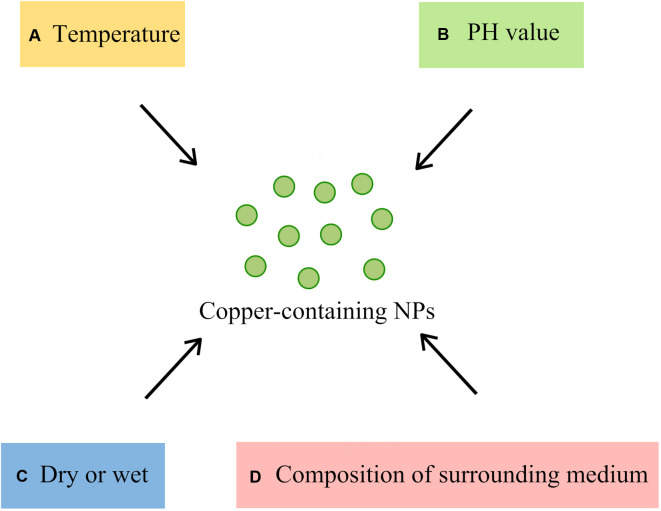
Ambient conditions affecting the antibacterial effect of Copper-containing NPs (**A**) temperature: the higher the temperature, the faster the sterilization; (**B**) PH value: the sterilization of acidic and alkaline conditions is faster than that of neutral conditions; (**C**) Dry or Wet: dry conditions kill bacteria faster than wet conditions; (**D**) composition of the surrounding medium: interaction of NPs with other molecules in the medium).

The ambient conditions of copper-containing nanoparticles are one of the factors that affect the antibacterial effect (Figure 3). The temperature and pH of the solvent affect the rate at which copper inhibits microorganisms. Studies by Sharan R et al. (17) showed that copper can cause the rapid inactivation of *E. coli* at higher temperatures and exhibit a faster inactivation at pH 6.0 and 9.0 than at pH 7.0 and 8.0. However, the effect of pH on bacterial inactivation is not as significant as that of temperature. The dissolution of copper ions is also an important part of the antimicrobial activity of copper-containing NPs. Usually, copper-containing NPs release more copper ions in an acid lysosomal environment than in a neutral environment (104, 105). Dry conditions bring about faster microbicidal effects to copper, as the contact killing caused by dry copper surfaces can kill microorganisms in a short amount of time. Tian et al. (106) demonstrated that the *Enterobacter* cell structure was severely degraded after exposure to the dry copper surface for 30 s. Moreover, compared to wet conditions, copper kills *Enterococcus* 80% to 90% faster under dry conditions (77). In the case of contact killing, the antimicrobial effect of copper is not related to the dissolution of copper but the copper content on the contact surface (107).

Besides, the interaction of copper-containing NPs with other molecules present in biological and environmental media may greatly affect the solubility, aggregation state, and surface properties of the NPs, resulting in changes of the toxicity (108). Studies by Badetti et al. (109) found that CuO NPs can react with some natural amino acids to affect their antimicrobial properties. L-Glutamic can be bonded to the surface of CuO NPs to enhance their antibacterial activity, while L-Asparaginase, L-Leucine, l-Phenylalanine, and L-Tyrosine can weaken the antibacterial activity of CuO NPs by forming complexes with copper ions. Ruparelia et al. (110) found that the chloride-containing nutrient media can promote the dissolution of Cu NPs, so as to release copper ions, which may be due to the interaction between the chloride ions and the oxide layer of the NPs.

### Doping modification

Doping modifications can modulate the interaction between NPs and microorganisms. Some studies have shown that doping other materials into Cu NPs and Cu_x_O NPs can improve their antimicrobial ability. For example, studies by Lv et al. (111) have shown that the doping of Mg, Zn, and Ce ions can promote the release of Cu^2+^ in the doped CuO NPs and promote their antibacterial activity. Studies by Malka et al. (112) have shown that Zn-doped CuO NPs can generate more ROS than pure CuO NPs or ZnO NPs and thus exert stronger antibacterial activity. After being exposed to *E. coli* and *Staphylococcus aureus* (*S. aureus*) for 10 min, the antibacterial activity of Zn-doped CuO NPs was 10,000 times greater than that of pure CuO NPs or ZnO NPs. Besides, other metal component of copper-containing bimetal NPs, such as iron, can promote the conversion of Cu^2+^ to more toxic Cu^+^ and Cu^3+^, which makes bimetallic iron-copper NPs exhibit a stronger antimicrobial activity than Cu NPs and iron NPs (Fe NPs) (113).

### Oxidation state

Copper has different antimicrobial properties under different oxidation states. The surface of pure copper is susceptible to oxidation and forms both CuO and Cu_2_O. Oxidative conditions (e.g., clean water in the air, upper hatched areas) contribute to the formation of CuO, while reducing conditions (e.g., the presence of organic matter and bacteria) contribute to the formation of Cu_2_O. These changes in oxidation state may affect the antibacterial properties of copper-containing NPs (105). Studies by O Akhavan et al. (114) have shown that Cu NPs exhibit greater antibacterial activity than CuO NPs, which may be attributed to the stronger electron-accepting ability and better electron transfer with bacteria of Cu NPs. The electron transfer between the negatively charged bacteria and the metal NPs is one of the effective mechanisms that cause the bacterial membrane rupturing and exerting antibacterial activity. Studies by Hans M et al. (115) have shown that Cu NPs and cuprous oxide NPs (Cu_2_O NPs) have strong contact killing activity against bacteria, while CuO NPs significantly inhibit contact killing. This difference is roughly related to the release of copper ions: pure copper releases the most copper ions, followed by Cu_2_O and CuO. Studies by Giannousi et al. (33) have also shown that the antibacterial activity of Cu_2_O NPs against a variety of Gram-negative and Gram-positive bacteria strains is stronger than CuO NPs. However, CuO NPs can induce higher ROS than Cu_2_O NPs, which is probably because CuO NPs can generate ROS through Haber-Weiss and Fenton type reactions, while Cu_2_O NPs can only generate ROS through Fenton type reactions. Moreover, CuO NPs have a higher degree of internalization and better antifungal activity at lower concentrations (24).

## Host tissue interaction of copper-containing NPs

Although copper-containing NPs are highly anticipated new materials, it is necessary to ensure their biosafety to human bodies (116–120). Copper is an essential trace element for the human body, participating in various kinds of physiological activities. Copper containing enzymes and transcription factors are essential for cellular integrity, energy production, signalling, proliferation, oxidation and radiation defence (121). The liver, brain, heart and kidneys have the highest copper concentration in the body, followed by the lungs, intestines and spleen.

**Figure 4:**
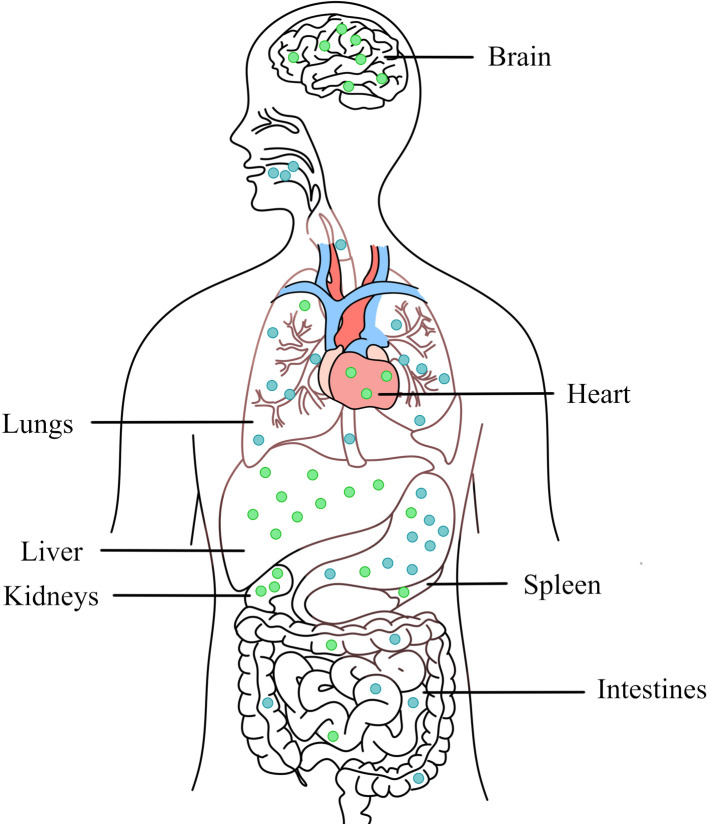
Main routes of transfer (blue) and accumulation (green) in organs of copper-containing NPs in the body through oral intake.

Research concerning acute or chronic toxicity of copper due to its deficiency or excess is growing rapidly and interest in the subject is pervasive (122–129). The four major routes of human exposure to engineered NPs include inhalation, dermal penetration, ocular exposure, and ingestion. Studies have shown that oral exposure of copper containing NPs in rats mainly accumulates in liver, kidney, stomach, intestine, lung, brain and blood, among which liver and kidney are the main organs most affected by Cu NPs (130, 131) (Figure 4). Exposure to NPs induces an inflammatory response and activates the immune system (132). The toxicity mechanism of copper-containing NPs to human cells is similar to that of microbial cells. Copper-containing NPs will dissolve and release copper ions, generate ROS, disrupt normal cellular functions and cause DNA damage. Changing the physicochemical properties of copper-containing NPs can change the induced toxic response/mechanism of action, such as size (aerodynamic, hydrodynamic), surface (surface area:mass ratio), chemical composition (core structure, surface functionalization, coatings), solubility (hydrophobic, hydrophilic) (133).

In dentistry, copper-containing NPs are expected to be used in restorative materials, prosthodontic materials, dental implants and orthodontic appliances. Researchers tried to explore safe forms to reduce toxicity of copper-containing NPs (116, 118). In fact, copper-containing NPs applied to dental materials rarely enter the body,and the concentration and morphological characteristics of copper-containing NPs can be controlled so that they will not be cytotoxic to normal cells (134). In addition, since copper-containing NPs can exert better microbial inhibition effect than copper native, addition amount of copper-containing NPs can be lower (44).

## Inhibitory effect of copper-containing nps on oral pathogenic microorganisms

Since microorganisms are less susceptible to resistance against metal antibacterial substances, the application of metals as antibacterial substances to control oral plaque has become a research hotspot. Studies have shown that copper-containing NPs can inhibit various oral pathogens, such as *S. mutans* (20, 21), *P. gingivalis* (23), and *C. albicans* (20, 24–26) (Table 1).

**Table 1:** The inhibitory ability of some copper-containing nanoparticles on oral microbes.

Nanoparticles (Diameter and morphology)	Test oral microbes	Anti-microbial test method	Antimicrobial efficiency	Mechanism of action	Reference
CuO NPs (40 nm)	*S.mutans* (PTCC 1683) *C.albicans* *Candida krusei* (*C. kruse*) *Candida krusei* (*C. glabrata*)	MIC (37°C, 48 h)	*S.mutans*: 1–10 µg/ml (MIC_50_) *C. albicans*, *C. kruse* and *C. glabrata*: 1000 µg/ml (MIC_50_)	Produce ROS	(20)
CuO NPs (39.87 nm, spherical)	Oral bacteria from the teeth crown surface	CuO NPs (10, 50, and 100 µg /ml) were treated with 10^6 ^CFU/ml bacterial cells (37°C,16 h)	10 µg/ml: 66% (NA agar plates) and 59% (MRS agar plates) inhibition of bacteria 50 µg/ml: Inhibits 82-92% of bacteria The EC_50_ values: 22.5 µg/ml (NA agar plates) 25 µg/ml (MRS agar plates)	Unclear	(21)
CuO NPs (18–20 nm, spherical)	*S.mutans* (700610)	Sonochemical coating of CuO NPs on artificial tooth surface treated with 10^7 ^CFU/ml bacterial cells (37°C, 24 h)	Biofilm formation is reduced by 70% Bacteria in the medium was not affected.	Produce ROS	(149)
CuCh NPs (131 ± 36 nm)	*S.mutans* (ATCC 25175)	MIC and MBC (37°C , 48h)	MIC: 35 µg/ml MBC: 60 µg/ml	Produce ROS Inhibit the activity of glucosyltransferase (GTF)	(42)
CuO (10.7 nm, nanobar) Cu_2_O (36 nm, nanocube)	*C. albicans* (ATCC 90028)	CuO and Cu_2_O were treated with 5 × 10^6 ^CFU/ml bacterial (37°C , 24 h)	The MIC of CuO and Cu_2_O is 150 µg/ml and 250 µg/ml respectively, and biofilm inhibitory concentration (BIC) for both NPs is 1 µg/ml	Produce ROS Destroy cell membranes Inhibits ergosterol and causes loss of virulence Inhibits yeast-to-hyphal transition	(24)
CuO Cu_2_O Ag + CuO composite [70% (w/w) Ag] (10–50 nm)	*P. gingivalis* (W83) *Prevotella intermedia* (*P. intermedia*, ATCC 25611) *Fusobacterium nucleatum* (*F. nucleatum*) subsp. nucleatum (ATCC 25586) *Aggregatibacter actinomycetemcomitans* (*A. actinomycetemcomitans*, ATCC33384)	CuO, Cu_2_O and Ag + CuO composite (100, 250, 500, 1,000 and 2,500 µg/ml ) were treated with 5 × 10^6 ^CFU/ml bacterial cells (37°C , 48 h)	For CuO: *P. gingivalis*: 500 µg/ml (MIC), 2500 µg/ml (MBC) *P. intermedia*: 250 µg/ml (MIC), 250 µg/ml (MBC) *F. nucleatum*: 250 µg/ml (MIC), 250 µg/ml (MBC) *A. actinomycetemcomitans*: 250 µg/ml (MIC), 250 µg/ml (MBC) For Cu_2_O: *P. gingivalis*: <100 µg/ml (MIC), <100 µg/ml (MBC) *P. intermedia*: <100 µg/ml (MIC), <100 µg/ml (MBC) *F. nucleatum*: <100 µg/ml (MIC), <100 µg/ml (MBC) *A. actinomycetemcomitans*:1,000 µg/ml (MIC), 1,000 µg/ml (MBC) For Ag + CuO composite: *P. gingivalis*: <100 µg/ml (MIC), <100 µg/ml (MBC) *P. intermedia*: <100 µg/ml (MIC), 100 µg/ml (MBC) *F. nucleatum*: 500 µg/ml (MIC), 500 µg/ml (MBC) *A. actinomycetemcomitans*: 250 µg/ml (MIC), 250 µg/ml (MBC)	Damage to cell membrane permeability Produce ROS	(23)
Fe doped CuO NPs (Rectangular shape assembled from approximately 23 µm microspheres and sheets with an average thickness of 150 nm)	*C. albicans*	Fe doped CuO NPs were treated with 1% overnight cultures of *C. albicans* (30°C, 24 h)	20 µg/ml: inhibited biofilm formation by 7.2%. 100 µg/ml: reduced the growth OD to 0.28 and inhibited the formation of biofilms by 76.4%	Release metal cations Combine with bacterial cells The Trojan horse mechanism	(26)
chitosan-copper NPs (The diameters of NPs containing 0.05, 0.1, 0.2 and 0.5 wt% chitosan are 50–300 nm, 50–270 nm, 5–50 nm and 2 nm, respectively)	*C. albicans*	chitosan-copper NPs (2,500 µg/ml) were treated with 1 × 10^5 ^CFU/ml fungal cells (37°C, overnight)	The inhibition rates of 0.05, 0.1, 0.2 and 0.5 wt% of NPs on *C. albicans* were 82.75, 82.2, 81.37 and 65.86%, respectively	The Trojan horse mechanism	(159)

**Table 2:** Antibacterial application of copper-containing nanoparticles in dentistry.

Nanoparticles (Diameter and morphology)	Oral materials description	Test oral microbes	Anti-microbial test method	Antimicrobial efficiency	Features	cytotoxicity	Reference
Cu NPs (40–60 nm, spherical) ZnO NPs (10–30 nm)	Add 5 /0.1 wt% and 5/0.2 wt% of ZnO / CuNp respectively to two commercial adhesives	*S. mutans* (ATCC 25175)	Disc diffusion method (37°C , 48 h)	Non-polymerized: Significantly higher antibacterial properties Polymerized: only the 5/0.2 groups showed significantly higher antibacterial properties.	Provides anti-MMPs properties without affecting its mechanical properties, thereby improving the integrity of the hybrid layer on caries-affected dentin.	Data not shown	(173)
Cu NPs (63–154 nm	Add 0.0075, 0.015, 0.06, 0.1, 0.5 and 1.0 wt% Cu NPs into the simplified etch and-rinse adhesive system	*S. mutans* (ATCC 25175)	Prepare disk-shaped adhesive specimens and place on BHI agar plates cultured with *S. mutans* (37°C , 96 h)	Improved antibacterial performance, with the highest antibacterial effect at 0.1, 0.5 and 1.0 wt%	Increase the immediate and 2-year bond strength of the resin-dentin interface, as well as the mechanical properties of the adhesive formulation after 2-years of water storage.	Data not shown	(174)
PAA-CuI NPs (20 nm–1.5 µm)	Mix PAA CuI powder with two commercial binder resins (XP Bond and Optibond XTR) to prepare PAA-CuI adhesive concentrations of 0.5 mg/ml or 1.0 mg/ml	*S. mutans* (ATCC 25175)	Resin composite discs are fabricated and coated with adhesive, and *S. mutans* is inoculated on the surface (37°C , 18 h; 37°C , 1y)	After18 h: Bacteria reduced by 99.99% (XP Bond) and 79.65% (XTR – 1.0 mg/ml) After 1y: Bacteria reduced by 99.99% (XP Bond)	Does not affect shear bond strength	No cytotoxicity ( human gingival fibroblast-like cells)	(176)
Cu NPs (Uncharacterized)	Add 0.01, 0.5 and 1 wt% of Cu NPs to the orthodontic composite	*S. mutans*	Disk-shaped adhesive specimens were prepared and placed in medium with *S. mutans* (37°C , 24 h)	Shows a significant antibacterial effect. The antibacterial effect was enhanced with the increase of NPs concentration	Does not affect shear bond strength	Data not shown	(180)
PAA-CuI NPs (59–88 nm)	0.263 wt% of PAA-CuI NPs were added to fluoroaluminosilicate glass powdersto generate Generation of PAA-CuI modified glass ionomer (GI) and PAA-CuI modified resin-modified glass ionomer (RMGI)	*S. mutans (ATCC 25175)*	Disk-shaped specimens were prepared,inoculated with 100 µl of *S. mutans* (1 × 10^8 ^cells/ml) on the surface (37°C , 18 h)	Reduce bacterial concentration by 99.999%	Does not affect mechanical properties Reduce the degradation of collagen in the dentin matrices	Data not shown	(181)
Cu NPs (10.87 nm)	Add1, 2, 3 and 4 wt% of Cu NPs to the glass ionomer cement	*S. mutans* (ATCC 25175) *Streptococcus sanguinis* (*S. sangius*, ATCC 10556)	Modified glass ionomer cement discs were prepared and placed in medium with *S. mutans* and *S. sanguinis* (1 × 10^6 ^cells/ml, 35°C, 48 h)	Significantly inhibited the growth of *S. mutans* and *S. sanguinis* (2–4 wt%)	Data not shown	After 72 h of exposure to modified glass ionomer (2–4 wt%) extract, the viability of human dental pulp fibroblasts remained above 68%.	(182)
CuO NPs (40–60 nm) TiO_2_ NPs (40–60 nm) ZnO NPs (20 nm) Ag NPs (50–60 nm)	The NPs were added to a water based-solution	*S. mutans* (PTC 1683) *S. sangius* (PTCC 1449)	Mix 50 ml of each sample with 50 ml of bacterial suspension (5 × 10^3 ^CFU) and incubate for 1 and 5 min	Both ZnONPs and CuONPs mouthwashes significantly reduced *S. mutans* after 1 and 5 min of exposure The colonies in all NPs groups after 5 min treatment was comparable to that of chlorhexidine	Data not shown	Data not shown	(183)
Cu NPs (50–100 nm)	The mussel-inspired dendritic polyglycerol (MI-dPG) surface coating doped with Cu NPs was prepared	*E. coli* *S. aureus* kanamycin-resistant *E. coli*	The sample was incubated with the various bacterial suspension for 24 h to detect the antibacterial rate. The same sample and its extract have been tested for long-term antibacterial activity against *E. coli* for 3d. After 40d of incubation, the sample was immersed in PBS or MilliQ for one month to test the durable antibacterial activity against *E. coli*. Anti-biofilm activity was assessed by incubating the sample with *E. coli* for 24 h.	The antibacterial rate against various bacteria is over 99.99%. In the three-day continuous antibacterial experiment, the antibacterial rates were 99.99%, 99.52% and 93.50%, respectively, and the antibacterial rate of the extract was less than 90%. After 40 days of culture, the Cu NPs in the coating can still effectively kill the attached bacteria and inhibit biofilm formation.	Excellent, long-lasting and broad-spectrum antibacterial properties with "attract-kill-release" characteristics	80% cell viability after 24 h (NIH/3T3 cells )	(195)
Cu NPs (20–30 nm, cubic geometry)	Deposition of Cu NPs on the surface of TiO_2_ nanotubes to form nCu–nT-TiO_2_ surface	*E. coli* (ATCC 25922) *S. aureus* (ATCC 6538)	Immerse the modified surface in the bacterial suspension (150 rpm, 37°C, 2 h)	100% reduction of surface adhesion of *E. coli* and *S. aureus*	Prevent early infection Enhance the adhesion of osteoblast Promote the colonization of bone cells	Data not shown	(196)
Cu NPs	Depositing Cu NPs on the surface of HA coating to obtain Cu-HA composite coatings	*E. coli* (JM109) *S. aureus* (ATCC 27217)	In the presence of coated titanium plates placed in bacterial suspension ( 1 × 10^7 ^cells/Ml, 37°C), monitor and measure several time points (0, 2, 4, 6, and 8 h) bacterial growth in the bacterial suspension.	The antibacterial rate gradually increases with the increase of copper content. The highest resistance rates to *E.coli* and *S.aureus* were 78% and 83%, respectively.	Enhance the osseointegration Provide a continuous antibacterial effect	Data not shown	(197)
ZnO NPs (45 nm) CuO NPs(37 nm) CuO-ZnO NPs	Deposited NPs on the orthodontic brackets	*S.mutans* (ATCC 35668)	Glue the brackets to the center of the buccal surface of each tooth. Add 1 ml of bacterial suspension (1.5 × 10^5 ^CFU/ml, 37°C ,180 shakes per minute), and detect the amount of bacteria at 0, 2, 4, 6 and 24 h.	Brackets oated with CuO NPs and ZnO-CuO NPs reduced the number of *S. mutans* to zero after 2 h.	Brackets coated with CuO NPs and ZnO-CuO NPs have excellent antibacterial effects on *S. mutans*	Data not shown	(198)

### S. mutans

It is well-known that *S. mutans* is the main pathogen of dental caries, which can adhere to the surface of tooth or dental prosthesis to form plaque biofilm, produces acid, and causes dental caries (135–139). Numerous studies have shown that copper can inhibit the growth of *S. mutans* and caries formation (140–143). In the case of extracellular high concentrations of copper, copper ions enter *S. mutans* cells and inhibit the transcription of glucosyltransferase (*gtf*) genes and glucan binding protein (*gbp*) genes to reduce cell adherence and biofilm biomass (144, 145).

**Figure 5:**
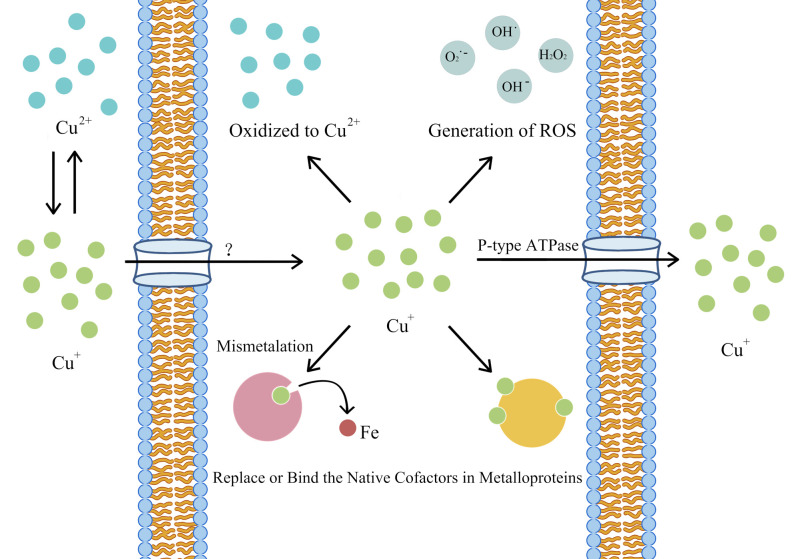
Copper transport system in bacteria.

Generally, bacteria have a “copper transport system” to cope with fluctuating copper ion concentrations in complex ecosystems and maintain copper homeostasis (Figure 5). *S. mutans* can tolerate extracellular high concentrations of copper through a conserved P-type ATPase, a copper-transport operon (146, 147). *S. mutans* can also oxidize intracellular Cu^+^ to less toxic Cu^2+^. Despite a certain degree of resistance to copper, copper-containing NPs can still effectively inhibit *S. mutans* through various mechanisms. Studies by Amiri et al. (20) have shown that the MIC_50_ value for CuO NPs with a size of 40 nm is 1–10 µg/ml for *S. mutans*, and higher concentrations of CuO NPs (100–1,000 µg/ml) can significantly inhibit bacterial growth. Similarly, Khan et al. (21) also demonstrated that CuO NPs at a size of 40 nm can significantly inhibit the growth of human oral pathogens (such as *S. mutans*), the extracellular polysaccharide production, and the multispecies biofilm formation at a concentration of 50 µg/ml. In another study, Eshed et al. (148) used the sonochemistry method to coat CuO NPs on the teeth surface, and the biofilm formation on the teeth coated with CuO NPs was significantly reduced by 70%. Similarly, Covarrubias et al. (42) synthesized hybrid NPs of chitosan-coated copper NPs (CuCh NPs), which can significantly inhibit the growth of *S. mutans* and significantly reduce biofilm formation.

### P. gingivalis

*P. gingivalis* is the main periodontal pathogen and is closely related not only to the occurrence of periodontal disease but also to the occurrence of systemic diseases, such as atherosclerosis, diabetes, and rheumatoid arthritis (149–153). Studies have shown that copper and copper alloys can inhibit the growth of *P. gingivalis* by contact killing, such as the Ti-Cu alloy, which can exert good antibacterial activity by killing the bacteria as well as reducing the activity of any surviving bacteria (154, 155). In addition, copper ions can inhibit the coaggregation of *P. gingivalis* with other bacteria, thereby reducing the accumulation of *P. gingivalis* in plaque biofilm and also reducing the pathogenicity and occurrence of periodontal disease (156). *P. gingivalis* is a Gram-negative bacterium with a lipopolysaccharide on the cell membrane surface that can prevent copper-containing NPs entering the cell. However, copper-containing NPs still have a significant inhibitory effect on *P. gingivalis*. Vargas-Reus et al. (23) found that the MICs of CuO NPs, Cu_2_O NPs and Ag + CuO composite with a size ranging between 10 nm and 50 nm were 500 µg/ml, <100 µg/ml and <100 µg/ml for *P. gingivalis*, respectively, suggesting that they have good antibacterial activity. Additionally, CuO NPs, Cu_2_O NPs and Ag + CuO composite has better antibacterial ability to *P. gingivalis* than ZnO NPs, TiO_2_ NPs and Ag NPs.

### C. albicans

*C. albicans* is the most common fungus in the oral cavity and is a conditional pathogen that often causes fungal infections in the elderly population or in denture patients. (157, 158) *C. albicans* can form a biofilm on the oral mucosa against external antifungal agents, which makes it pathogenic (159). Copper-containing NPs have also exhibited considerable antimicrobial activity against *C. albicans*. Padmavathi et al. (24) found that both CuO NPs and Cu_2_O NPs have fungal inhibitory activity. They can destroy the cell membrane of *C. albicans* by inhibiting the production of ergosterol, which lead to the loss of virulence. They can also alter the expression of the genes involved in the morphogenesis of *C. albicans*. CuO NPs can inhibit mycelial growth, while Cu_2_O NPs can distinctively inhibit morphological switching. Moreover, CuO NPs have a stronger inhibitory effect on *C. albicans* than Cu_2_O NPs and exhibit better antifungal activity at a low concentration. Amiri et al. (20) found that CuO NPs with a size of 40 nm couldreduce the growth of *C. albicans*, *Candida krusei*, and *Candida glabrata*, and the MIC_50_ value of CuO NPs was 1,000 µg/ml for these three species of oral Candida. Pugazhendhi et al. (26) synthesized Fe-doped CuO using a sol-gel method, which has a rectangular shape and agglomerates at an average size of 21 nm. The Fe-doped CuO has excellent antimicrobial and anti-biofilm properties to *C. albicans*, which can reduce the growth OD of *C. albicans* to 0.28 at 30°C for 24 h and reduce the biofilm by 76.4% at a concentration of 100 µg/ml. In another study, Lara et al. (159) synthesized chitosan-copper NPs with a size between 2 nm and 350 nm and proved that they had good antimicrobial activity against C. albicans.

## Application of copper-containing NPs in dentistry

**Figure 6:**
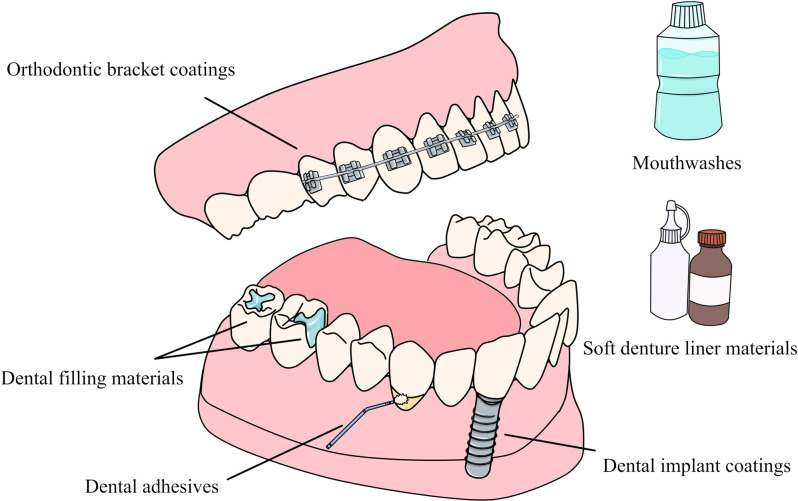
Application of copper-containing NPs in dentistry.

Copper-containing NPs can be applied to various aspects of dentistry. Applying NPs to the surface of dental materials or incorporating them in dental materials can not only impart different antibacterial activity to the material, but also improve or maintain the mechanical properties of the material (22, 160, 161). When applied to dental materials, they can also play a variety of beneficial roles by inhibiting metalloproteinases (MMPs) (146). Many current studies have synthesized different types of copper-containing NPs that can be used for dental materials, including dental adhesive es and filling materials, implant and bracket coatings, etc. (Figure 6) and (Table 2).

### Dental adhesives

Many recent studies have shown that copper-containing NPs in dental adhesives can not only effectively inhibit bacteria but also improve the performance of the adhesive. Copper ions released by copper-containing NPs can be used as an effective dentin metalloproteinase (mainly on the Matrix metalloproteinase's subtypes −2 and −9) inhibitor, which can stimulate the secretion of the tissue inhibitors of MMPs (162). Matrix metalloproteinase 2 (MMP-2) is involved in the destruction of periodontal tissue and the development of oral squamous cell carcinoma, and it also plays an important role in the destruction of dentin during the progression of caries (162–165). MMPs can also mediate the degradation of adhesives, while inhibiting MMP can increase the longevity of the adhesive-hard tissue interface and improve the bonding effect of adhesives (166–169). Besides, MMPs inhibitors can prevent dental caries, reduce dentinal caries progression, and promote remineralization (170–172). Studies by Gutiérrez et al. (173) have shown that the addition of Cu NPs and ZnO NPs to the universal adhesive system can provide the adhesive with antibacterial activity against *S. mutans* and anti-MMPs properties without affecting its mechanical properties, thereby improving the integrity of the hybrid layer on caries-affected dentin. The addition of copper-containing NPs to the adhesive may also improve its mechanical properties. Vidal Oet al. (174) incorporated copper nanoparticles (CuNp) into a universal adhesive and applied it to dentin surfaces. The addition of copper nanoparticles can significantly enhance the antibacterial activity of the resin-dentin interface, showing higher bond strength and mechanical properties, even under cariogenic challenges. Javed et al. (175) incorporated CuO NPs and CuO-chitosan NPs into dentin adhesives, which can significantly inhibit *Lactobacillus acidophilus* (*L. acidophilu*) and *S.mutans* and effectively treat secondary caries. The addition of NPs also improved the mechanical properties, water absorption and solubility of the adhesive without affecting the shear bond strength.

Many studies have also shown that adhesives with copper-containing NPs can exhibit long-lasting and effective antimicrobial effects. For example, studies by Gutiérrez et al. (176) have shown that the addition of Cu NPs at a concentration of 0.1 wt% in the adhesive system can provide antibacterial properties without reducing the mechanical and optical properties of the adhesive formulations. Moreover, compared with copper-free adhesives, copper-containing adhesives can significantly reduce the dentin degradation of the resin-dentin bonded interfaces dentin after two years of water storage. In addition, a sufficient concentration of copper still exists in the adhesive interface, which can exert anti-MMPs effects. In another study, Jun et al. (177) synthesized novel copper-doped bioactive glass NPs (CuBGn NPs) and added them to the resin-dentin adhesive system. Although there are no antibacterial experiments to prove its antibacterial properties, the adhesive can release up to 0.5 ppm copper ions over a 28-day period, which is sufficient to deactivate MMPs, promote remineralization, and extend the longevity of resin-dentin interfaces dentin regeneration.

Other studies have shown that adding copper-containing NPs to adhesives will not cause additional cytotoxicity to the pulp or oral soft tissues. For example, Sabatini et al. (178) synthesized polyacrylic acid coated copper iodide NPs (PAA-CuI NPs) and incorporated them into adhesives. After ageing for 18 h or one year, the adhesive can exert effective antibacterial effects without affecting the bond strength and cytotoxicity. A study by Matos et al. (179) also confirmed that the addition of 0.1 wt% Cu NPs to the adhesive can improve the clinical performance of universal adhesive systems in non-carious cervical lesions without increasing cytotoxicity.

Moreover, Cu NPs and CuO NPs can also be added to orthodontic adhesives to exert a certain antibacterial effect. Studies have shown that the addition of Cu NPs can significantly improve the material shear bond strength, while the addition of CuO NPs will not adversely affect the shear bond strength (180).

### Dental filling materials

Copper-containing NPs can also be used in dental filling materials. Studies by Renné et al. (181) have shown that the incorporation of polyacrylic acid coated copper NPs (PAA-CuI NPs) into glass ionomer-based materials can improve their antibacterial properties and reduce collagen degradation without affecting the mechanical properties, which can help increase the longevity of adhesive restorations. Aguilar-Perez et al. (182) synthesized copper-containing NPs composed of metallic copper and cuprous oxide and added them to commercial glass ionomer cement, confirming that they can inhibit oral anaerobic bacteria strains. In addition, the glass ionomer cement doped with copper-containing NPs has no cytotoxic effect and will not damage the dental pulp.

### Antimicrobial coatings

NPs can be used to control the formation of microbial biofilms in the oral cavity, which allows them to be incorporated into coatings and applied to a variety of dental materials (183, 184). Although dental implants have a high success rate, there are still failures. Poor osseointegration and infection are important reasons for implant failure (185). Coatings containing copper-containing NPs are commonly used in dental and orthopedic implants to increase their success rates by improving bone binding capacity and reducing the incidence of post-surgery infections (186, 187). Copper-containing NPs reduce the formation of biofilms on the surface of titanium implants. Moreover copper is involved in enzyme-based processes for bone metabolism and stimulates the formation of new blood vessels, which, in turn, reduces implant-related infections (90). Therefore, coating the titanium surface of the implant with copper-containing NPs can reduce the use of prophylactic antibiotics, which may cause the development of antibiotic resistant strains (188–190). Besides, the incorporation of an appropriate amount of Cu NPs on the implant surface not only has no cytotoxicity to endothelial cells and osteoblasts but also promotes osteoblast proliferation and adhesion as well as extracellular matrix mineralization (191).

Many studies have confirmed that the coatings containing copper-containing NPs can exert antimicrobial activity and be used in dentistry (192–194). For example, Li et al. (195) prepared an antibacterial coating material based on mussel-inspired dendritic polyglycerol embedded with Cu NPs, which not only has a bacteriostatic rate of over 99.99% against *S. aureus*, *E. coli*, and kanamycin-resistant *E. coli*, but also can exert effective long-term and durable antibacterial properties against *E. coli*. Rosenbaum et al. (196) prepared copper nanocubes with an average size of 20 nm on the surface of TiO nanotubes. This copper derived TiO surfaces could cause the death of *E. coli* and *S. aureus* and exert a powerful bactericidal ability. Ghosh et al. (197) used a two-stage electrochemical method to synthesize copper-hydroxyapatite (Cu-HA) composite coatings on titanium surfaces, which can slowly release copper ions while enhancing implant osseointegration to provide a sustained bacteriostatic effect. In addition, CuO NPs can be coated on the surface of orthodontic brackets. Studies have also shown that the CuO NPs and ZnO-CuO NPs coatings on the surface of orthodontic brackets have stronger antibacterial effects on *S. mutans* than ZnO NPs coatings (198).

### Mouthwashes

Copper-containing NPs also have the potential to be added to mouthwashes for antimicrobial action. In one study, CuO NPs were prepared in colloidal solutions as mouthwashes, and it was found that, although not as good as chlorhexidine mouthwash, CuO NPs can also have a certain antibacterial effect on *S. mutans* (199).

### Soft denture liners

The intrinsic porosity of soft denture pads facilitates the adhesion and colonization of microorganisms and promotes the formation of biofilms. A study by et al. (200) showed that the incorporation of CuO NPs at a concentration of 500 µg/ml into soft denture liners exerted an effective prevention of oral microbial infection. The biofilm inhibition rates of soft denture liners containing CuO NPs against *C.albicans*, *Streptococcus sobrinus* (*S. sobrinus*), *S. mutans*, and *Streptococcus salivarius* (*S. salivariu*) were 75%, 66%, 30%, and 60%, respectively.

## Conclusion

Many current studies indicate that copper-containing NPs can be used in dentistry due to their antimicrobial and anti-biofilm properties. Copper-containing NPs are a new type of ideal antimicrobial material, which can inhibit or kill a variety of oral pathogenic microorganisms without causing microbial resistance, and can also produce a certain degree of beneficial effects on oral tissues. Various forms of copper-containing NPs are still being explored for use in dental filling materials, prosthetic devices and implant coatings, and oral antimicrobial agents. However, many of these studies have been performed under *in vitro* conditions, and further *in vivo* studies are needed to assess their safety and clinical efficacy.
